# Post pancreatitis/cholecystectomy gluten intolerance 

**Published:** 2018

**Authors:** Ivan Tang, George MacFaul, Ravi Madhotra, Kamran Rostami

**Affiliations:** *Department of Gastroenterology, Hepatology and Nutrition, Milton Keynes University Hospital, United Kingdom *

**Keywords:** Gluten sensitivity, Non-coeliac, Post-pancreatitis, Post-cholecystectomy gluten intolerance

## Abstract

This case report describes the journey of a patient who suffered from life-limiting gastrointestinal symptoms after an acute bout of pancreatitis following ERCP for cholelithiasis bile following a ductal stone, and subsequent cholecystectomy. She was diagnosed and treated for IBS with medication without significant improvement. On implementation of a simple gluten and lactose exclusion diet she recovered to her premorbid state, and trials of gluten challenge triggered flares of symptoms. This case report will go on to discuss current evidence for use of gluten and lactose exclusion diets in some gluten sensitive patients misdiagnosed with IBS.

## Introduction

 Non-coeliac gluten sensitivity (NCGS) and lactose intolerance may occur after cholecystectomy, pancreatitis or gastroenteritis (1). None of these conditions are associated with serological or radiological markers (2) and they are currently labelled as irritable bowel syndrome (IBS). The treatment of these conditions can differ significantly and early consideration of gluten/lactose intolerance can lead to better management of symptoms, with reduced need for outpatient follow-up, invasive investigations and expensive symptomatic treatment (3). In this report, a case misdiagnosed as IBS shall be presented.

## Case report

A 41-year-old lady presented with several years history of abdominal pain and reflux. Her past medical history includes anti-TPO positive hypothyroidism treated with thyroxine, heavy menstrual bleeding and she had a BMI of 27kg/m2. In January 2013, two years prior to her clinic encounter, she developed sudden-onset abdominal pain and was diagnosed with cholecystitis which was investigated by ERCP. Two weeks later she developed pancreatitis from which she recovered well. She went on to have a laparoscopic cholecystectomy in February 2013. Since the procedures, she developed severe gastro-oesophageal reflux, abdominal pain, bloating and diarrhoea alternating with constipation. Clinical examination was mostly unremarkable except mild tenderness in both the right and left iliac fossae without organomegaly or palpable masses.

Her investigations in the gastroenterology clinic were negative for coeliac disease with negative endomysial antibodies. The gastroscopy revealed only reactive gastritis negative for Helicobacter pylori and more importantly, normal duodenal histology. The colonoscopy and liver ultrasound detected no abnormalities except multiple liver cysts. Her blood results were all within normal limits, including full blood count, urea and electrolytes, liver function testing, inflammatory markers, vitamin D, haematinics and thyroid hormones.

She was given a clinical diagnosis of IBS, but pharmacological treatment for this with Mebeverine and Hyoscine Butyl bromide did not produce much symptomatic change. After multiple follow-ups with several gastroenterologists and a dietician, she was given a trial of a gluten-free diet (GFD) and exclusion of lactose. These measures gave the patient significant symptomatic control, albeit without becoming completely asymptomatic. Re-introduction of gluten caused symptomatic flares. 

## Discussion

NCGS is a disorder clinically similar to coeliac disease, with intestinal and extra-intestinal symptoms caused by ingestion of gluten, however without the serological markers associated with coeliac disease (4). The pathophysiology has yet to be defined although various mechanisms, including immunological (5) and genetic predispositions (6), have been proposed. Diagnosis, according to the Salerno Expert Criteria, is made clinically if there is greater than thirty percent symptomatic improvement on a GFD with return of symptoms with gluten challenge (7).

As with all treatments there are risks and benefits. A GFD can cost significantly more than a normal diet (8, 9) and some studies suggest it may also cause nutritional deficiencies (8) while Lis et al. (10) claim that a GFD does not impact athletic performance. Arguably, symptomatic benefit outweighs the costs if the patient feels that there is subjective improvement. Treating NCGS incorrectly as IBS may lead to inadequate symptom control and therefore costs the healthcare system through the failing treatments and sick days (11).

A 2016 randomised double-blinded placebo-controlled trial by Zanwar et al. (12) suggested that gluten challenges after 4 weeks of a GFD on patients with negative serological markers and negative histology for coeliac disease had statistically significant effect on abdominal pain, bloating and tiredness when compared with a placebo gluten challenge (12). Another randomised double-blinded placebo-controlled trial in 2015 by Shahbazkhani et al. (13) yielded very similar and statistically significant results. The study population was selected from a group of patients who reported IBS-like symptoms, and were later narrowed down to those who responded well to a GFD, which would support the similarity in presentation of NCGS and IBS.

**Figure 1:**
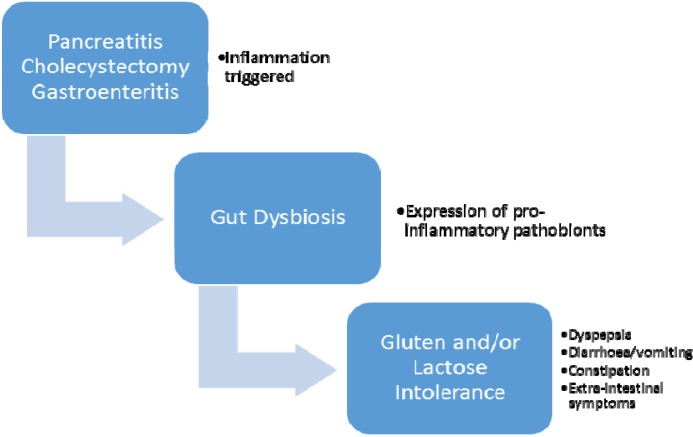
Gut dysbiosis triggered by gastroenteritis, pancreatitis, or cholecystectomy can promote inflammation through expansion of pro-inflammation pathobionts. This may result in lactose and gluten intolerance

Lactose and gluten intolerance after cholecystectomy could be explained by a postsurgical inflammatory response (14) and dysbiosis which can cause secondary lactase and peptidase deficiencies by way of microscopic and sub-microscopic (15) damages to intestinal epithelium (16). Changes in epithelial barrier function and permeability in NCGS are controversial in the current literature (Figure 1).

NCGS, has a similar presentation to coeliac disease and it could be disabling in some cases (17). Unlike IBS, it has many extra intestinal associations and it should not be labelled with IBS. 

Patients presenting with IBS-like symptoms after pancreatitis or cholecystectomy may respond to gluten/lactose exclusion diets. Persevering with IBS treatment may be in vain in those who have actually developed NCGS or lactose intolerance post pancreatitis or cholecystectomy, and may lead to prolonged reduced quality of life, reduced patient satisfaction and a greater cost to the healthcare system. An exclusion diet may be a simple and cost-effective treatment for these patients, and those using this strategy can also aid earlier diagnosis leading to better patient outcomes.

## Conflict of interests

The authors declare that they have no conflict of interest.
